# On the Office of the Surgeon-Apothecary

**Published:** 1813-04

**Authors:** 


*To the Editors of the Medical and Physical Journal.*

GENTLEMEN,

I HAVE with great pleasure, and I hope with some degree of benefit, read your excellent Journal, since its first commencement; but must confess I observed some strictures, in a late Number, by Censorinus, upon the Surgeon-Apothecaries, which I thought not only uncandid, but extremely illiberal.

So far from agreeing with Censorinus, that a degree of jealousy subsists between the apothecaries and physicians, I am of a very different opinion. It has been invariably my practice, in every case where danger was in the least suspected, to recommend calling in some physician, (always leaving the person called in to the patient's own choice.) This plan, by dividing the responsibility, has always afforded me, as it must every one else, peculiar satisfaction; and it is a fact well known, that patients take their medicines more cheerfully and more *plentifully* when recommended by the physician.

It has been my lot, within the last few years, to have the aid of an eminent and scientific physician called in to some as dangerous, perplexing, and refractory, cases, as could possibly be met with; and, so far from any malevolence subsisting between us, I have received considerable information from his sensible conversation; and I am sure his humane and generous mind would have spurned at the idea of calumny or detraction.

Censorinus lays a particular stress upon apothecaries attending and prescribing for the sick, thereby depriving the physician of his emoluments. I would ask Censorinus, who, in remote situations, where a physician is perhaps at a distance tance of forty or fifty miles, but the apothecary must prescribe for those who require medical assistance?—I could also inform him, that several surgeon-apothecaries, within my knowledge, are possessed of such precision in detecting diseases, and such neat and judicious modes of prescribing as would do honor to a diploma. In an obscure country village in Yorkshire, Censorinus perhaps may have heard of a person who formerly practised as a surgeon-apothecary, having for several years deservedly ranked as one of the first mathematicians in the kingdom; yet, was such an elevated character now in practice, he must patiently bear the opprobrious epithet of a "sleek and pampered apothecary," according to the denomination Censorinus is pleased to give us. Indeed, from the general tenor of Censorinus's communication, I am inclined to think, that he wishes, with one fell sweep, to brush from the face of the earth an industrious and meritorious class of society.

With respect to the assertion of Censorinus, that "the apothecaries are already in the undisputed possession of three branches of the profession out of four," nothing can be more incorrect. In country towns it is a well-known fact, that physicians not only practise surgery and midwifery, but likewise vaccinate children, phlebotomise, and extract teeth. I know, from sad and woful experience, that the surgeon-apothecaries are, without exception, a most oppressed class of people. While provisions, and every other necessary of life, are more than double the price they were formerly, the charges of the apothecary remain stationary, and they are the only class of society who receive no remuneration for their time and trouble. Suppose, for instance, which has often been the case with me, I call twice a-day upon a patient, for five or six months—my Christmas bill amounts to fifteen or twenty shillings—does this paltry sum, I would ask any candid person, repay me for the time and fatigue the patient's case seemed to require?

Before taking leave of Censorinus, I will make one candid appeal to his conscience: if, when we see the clergyman and weaver, forgetting the sphere of action they ought to occupy, turning vaccinators, bone-setters, &c.; when we see old wives daily usurping the place of the regular apothecary; when we daily hear that his Majesty's liege subjects are quickly hurried into the other world by the dangerous practice of empirics; if, I say, when abuses of such enormous magnitude exist, it is surely high time to solicit the interference of the legislature. In my opinion, the praiseworthy praiseworthy exertions of the Committee of Apothecaries deserve the thanks of the public; and, should the intended Bill meet with the encouragement it justly deserves, their memory ought to be held by posterity with the most grateful remembrance.—I am, in truth,

*A sleek, but not a pampered, Apothecary.*

*Kirkham, Feb.* 9, 1813.

